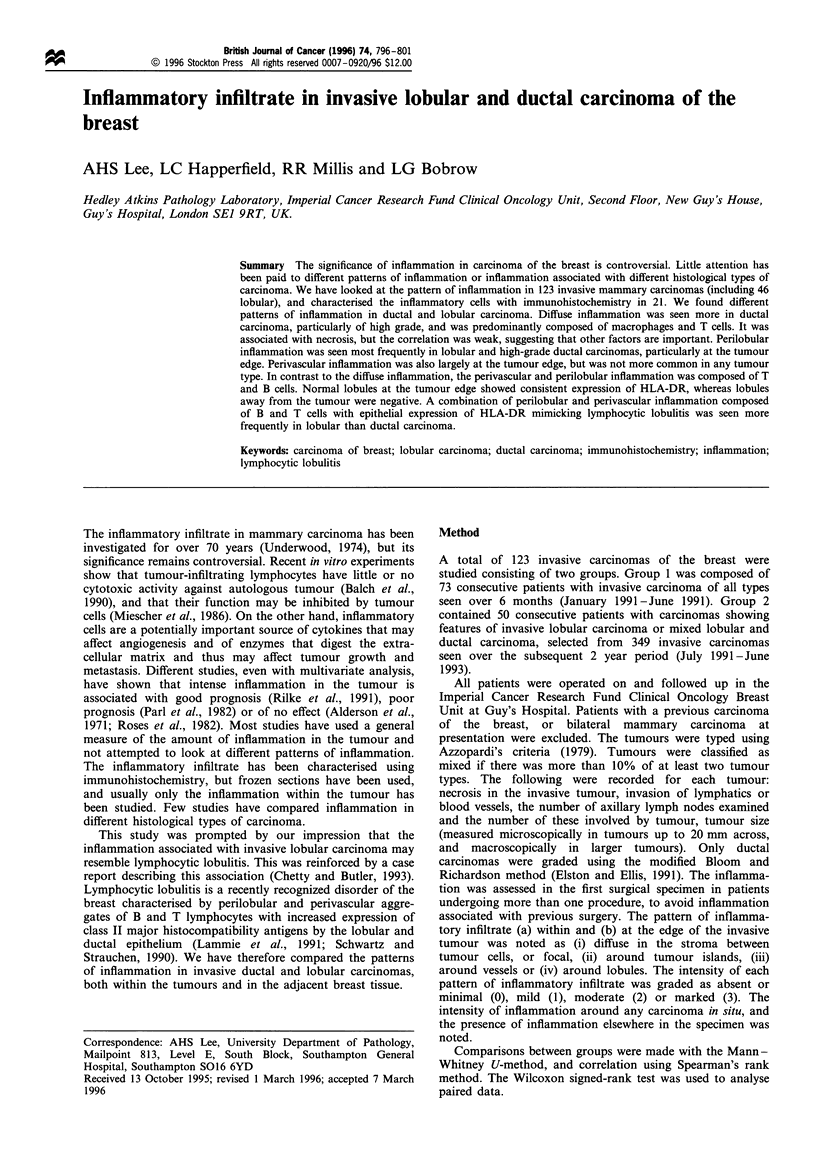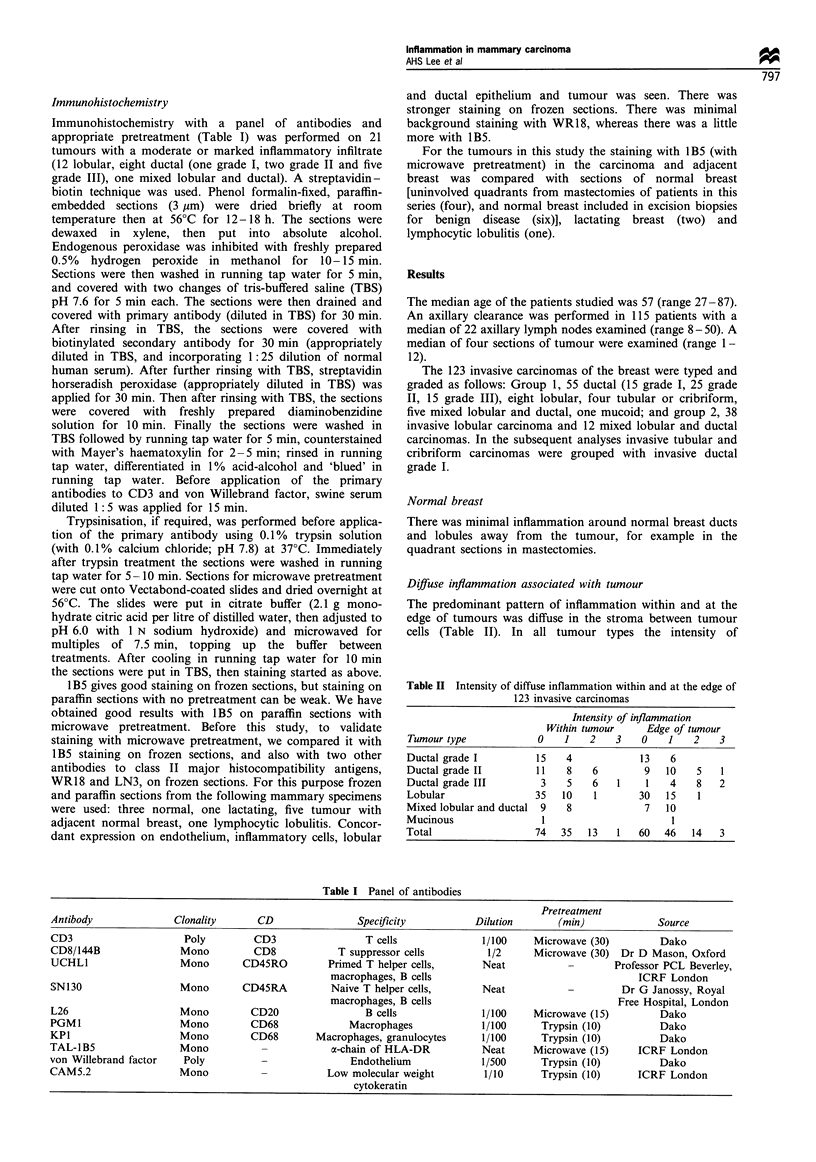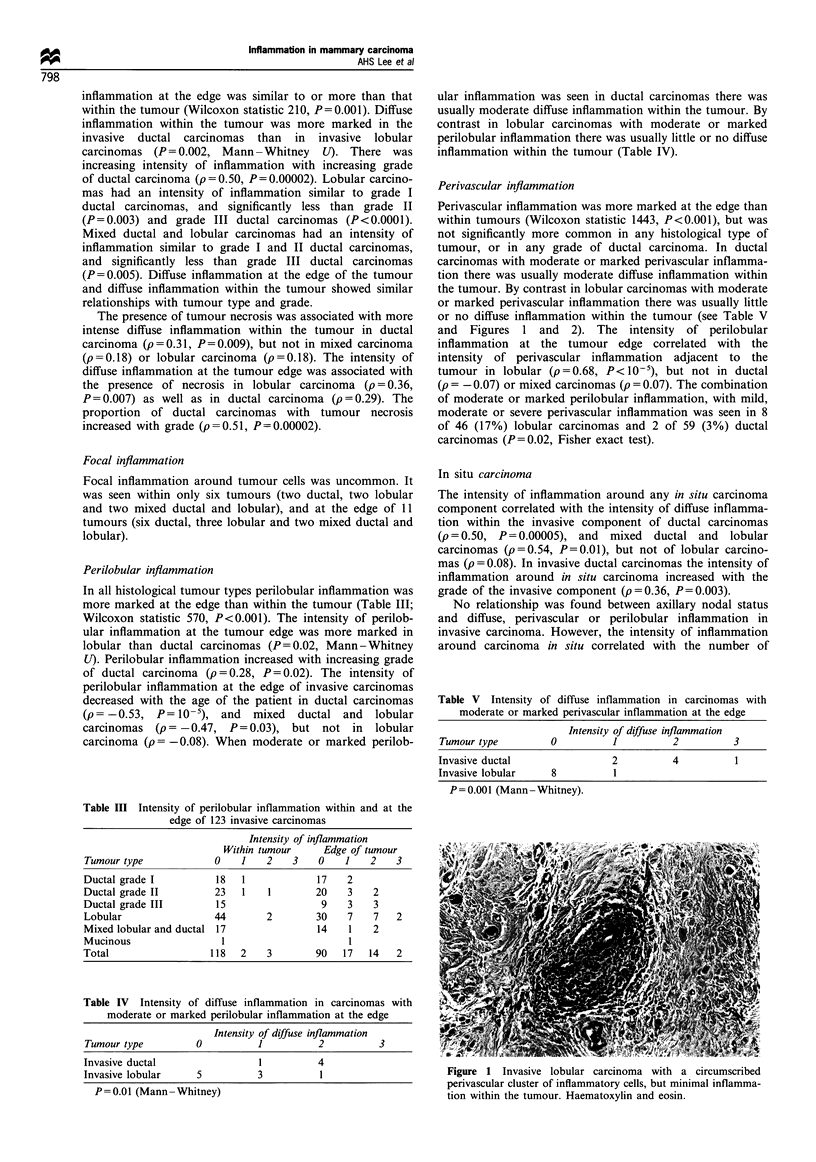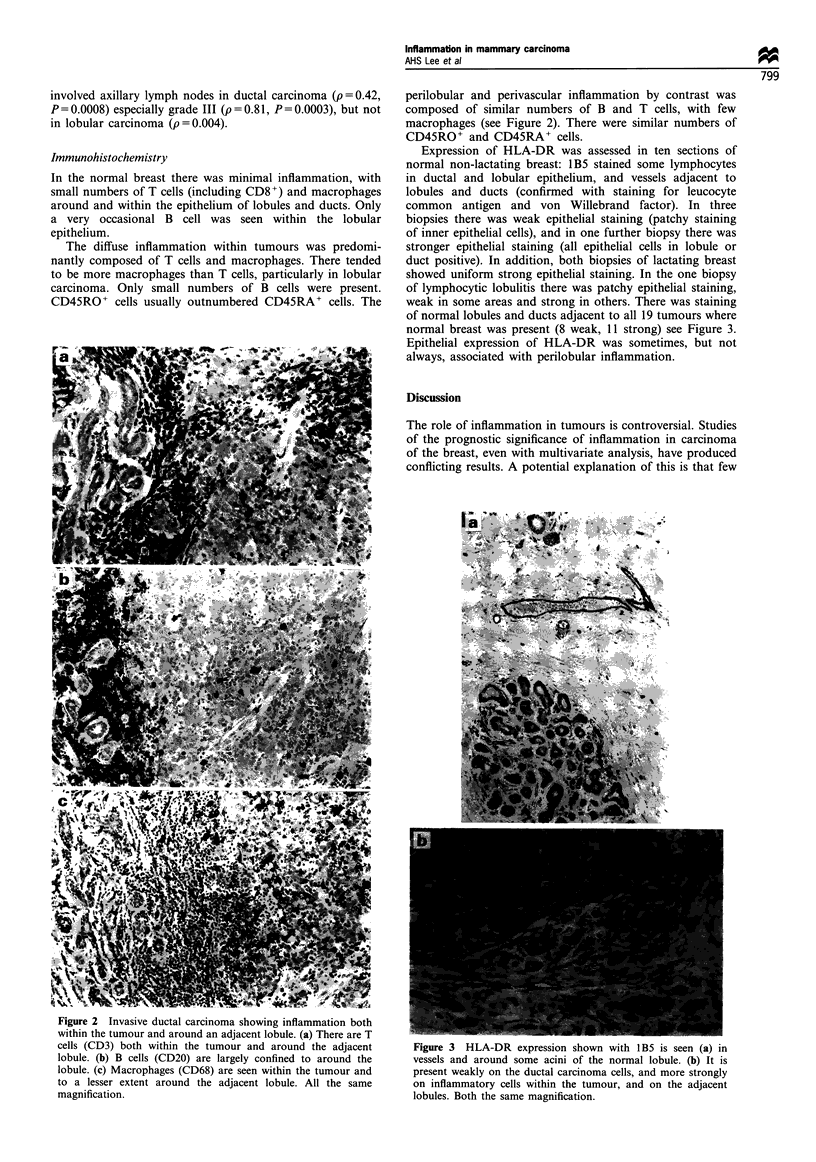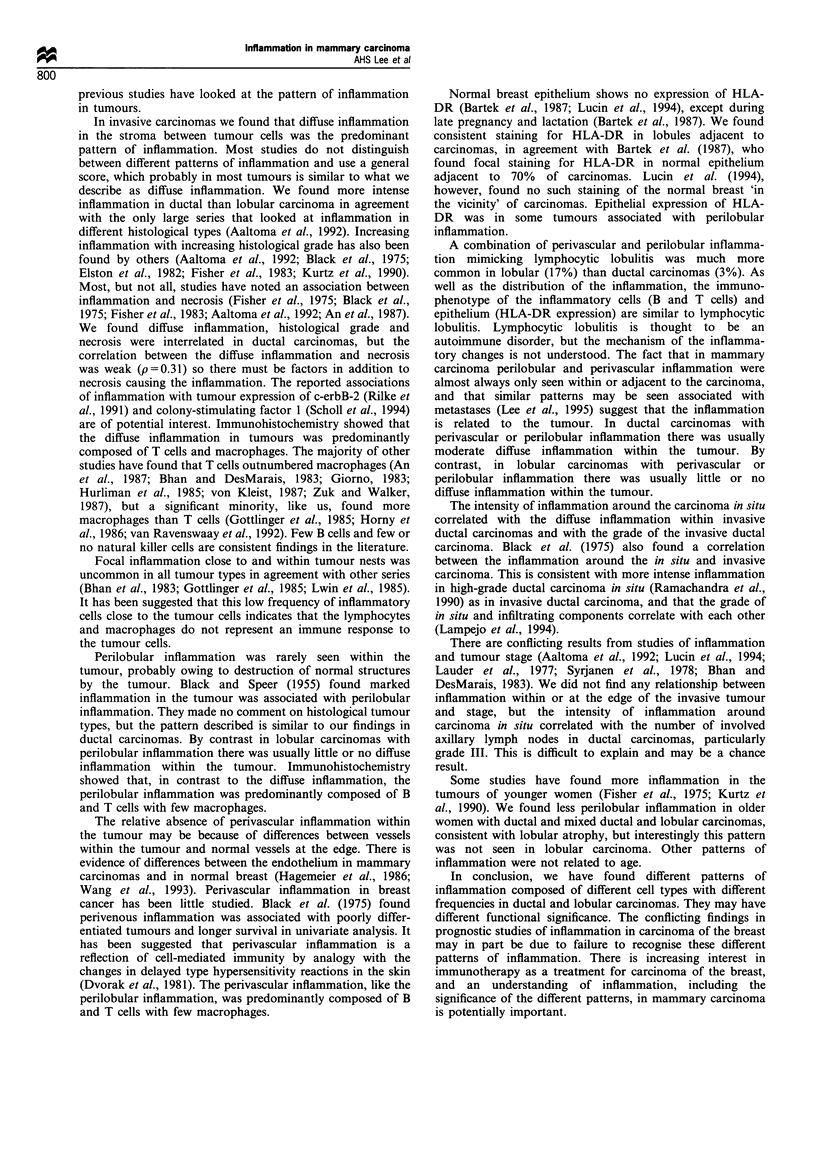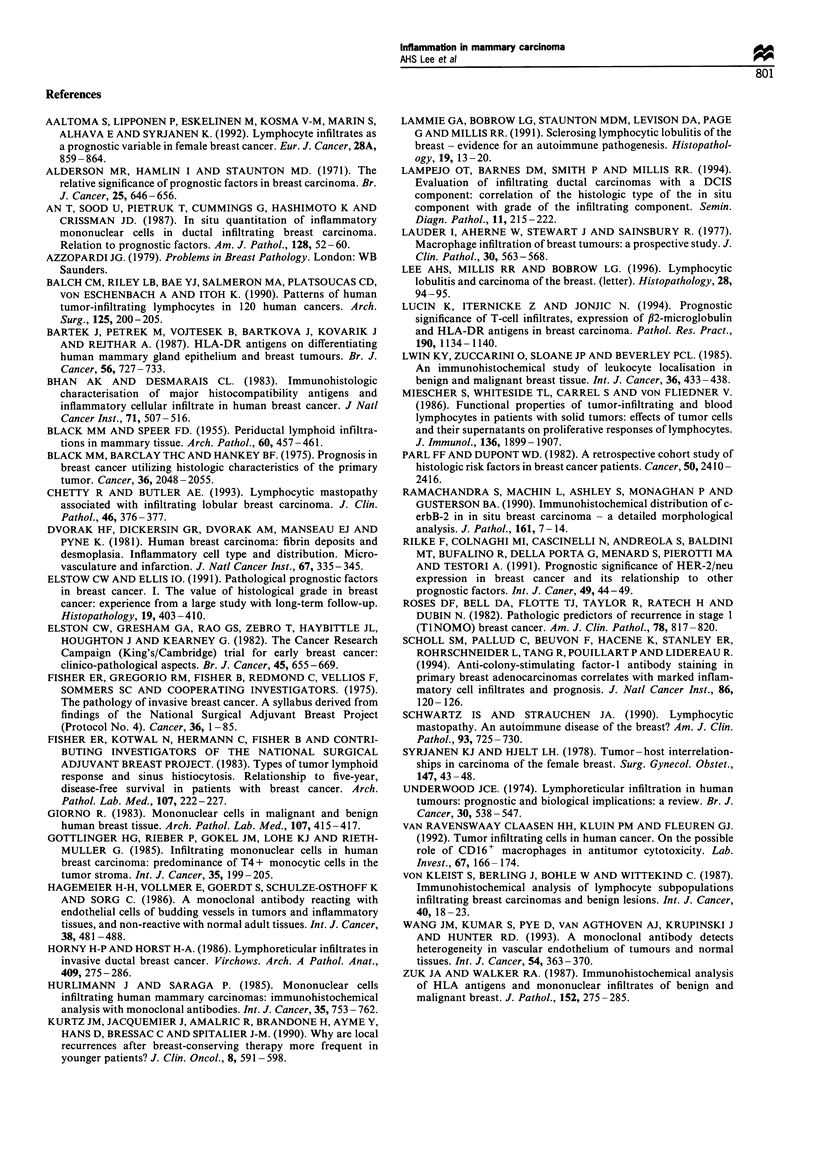# Inflammatory infiltrate in invasive lobular and ductal carcinoma of the breast.

**DOI:** 10.1038/bjc.1996.438

**Published:** 1996-09

**Authors:** A. H. Lee, L. C. Happerfield, R. R. Millis, L. G. Bobrow

**Affiliations:** Hedley Atkins Pathology Laboratory, Imperial Cancer Research Fund Clinical Oncology Unit, London, UK.

## Abstract

**Images:**


British Journal of Cancer (1996) 74, 796-801
© 1996 Stockton Press All rights reserved 0007-0920/96 $12.00

### Inflammatory infiltrate in invasive lobular and ductal carcinoma of the breast

AHS Lee, LC Happerfield, RR Millis and LG Bobrow

*Hedley Atkins Pathology Laboratory, Imperial Cancer Research Fund Clinical Oncology Unit, Second Floor, New Guy's House, Guy's Hospital, London SE1 9RT, UK.*

**Summary** The significance of inflammation in carcinoma of the breast is controversial. Little attention has been paid to different patterns of inflammation or inflammation associated with different histological types of carcinoma. We have looked at the pattern of inflammation in 123 invasive mammary carcinomas (including 46 lobular), and characterised the inflammatory cells with immunohistochemistry in 21. We found different patterns of inflammation in ductal and lobular carcinoma. Diffuse inflammation was seen more in ductal carcinoma, particularly of high grade, and was predominantly composed of macrophages and T cells. It was associated with necrosis, but the correlation was weak, suggesting that other factors are important. Perilobular inflammation was seen most frequently in lobular and high-grade ductal carcinomas, particularly at the tumour edge. Perivascular inflammation was also largely at the tumour edge, but was not more common in any tumour type. In contrast to the diffuse inflammation, the perivascular and perilobular inflammation was composed of T and B cells. Normal lobules at the tumour edge showed consistent expression of HLA-DR, whereas lobules away from the tumour were negative. A combination of perilobular and perivascular inflammation composed of B and T cells with epithelial expression of HLA-DR mimicking lymphocytic lobulitis was seen more frequently in lobular than ductal carcinoma.

**Keywords:** carcinoma of breast; lobular carcinoma; ductal carcinoma; immunohistochemistry; inflammation; lymphocytic lobulitis

The inflammatory infiltrate in mammary carcinoma has been investigated for over 70 years (Underwood, 1974), but its significance remains controversial. Recent *in vitro* experiments show that tumour-infiltrating lymphocytes have little or no cytotoxic activity against autologous tumour (Balch *et al.*, 1990), and that their function may be inhibited by tumour cells (Miescher *et al.*, 1986). On the other hand, inflammatory cells are a potentially important source of cytokines that may affect angiogenesis and of enzymes that digest the extracellular matrix and thus may affect tumour growth and metastasis. Different studies, even with multivariate analysis, have shown that intense inflammation in the tumour is associated with good prognosis (Rilke *et al.*, 1991), poor prognosis (Parl *et al.*, 1982) or of no effect (Alderson *et al.*, 1971; Roses *et al.*, 1982). Most studies have used a general measure of the amount of inflammation in the tumour and not attempted to look at different patterns of inflammation. The inflammatory infiltrate has been characterised using immunohistochemistry, but frozen sections have been used, and usually only the inflammation within the tumour has been studied. Few studies have compared inflammation in different histological types of carcinoma.

This study was prompted by our impression that the inflammation associated with invasive lobular carcinoma may resemble lymphocytic lobulitis. This was reinforced by a case report describing this association (Chetty and Butler, 1993). Lymphocytic lobulitis is a recently recognized disorder of the breast characterised by perilobular and perivascular aggregates of B and T lymphocytes with increased expression of class II major histocompatibility antigens by the lobular and ductal epithelium (Lammie *et al.*, 1991; Schwartz and Strauchen, 1990). We have therefore compared the patterns of inflammation in invasive ductal and lobular carcinomas, both within the tumours and in the adjacent breast tissue.

Correspondence: AHS Lee, University Department of Pathology, Mailpoint 813, Level E, South Block, Southampton General Hospital, Southampton SO16 6YD
Received 13 October 1995; revised 1 March 1996; accepted 7 March 1996

### Method

A total of 123 invasive carcinomas of the breast were studied consisting of two groups. Group 1 was composed of 73 consecutive patients with invasive carcinoma of all types seen over 6 months (January 1991–June 1991). Group 2 contained 50 consecutive patients with carcinomas showing features of invasive lobular carcinoma or mixed lobular and ductal carcinoma, selected from 349 invasive carcinomas seen over the subsequent 2 year period (July 1991–June 1993).

All patients were operated on and followed up in the Imperial Cancer Research Fund Clinical Oncology Breast Unit at Guy's Hospital. Patients with a previous carcinoma of the breast, or bilateral mammary carcinoma at presentation were excluded. The tumours were typed using Azzopardi's criteria (1979). Tumours were classified as mixed if there was more than 10% of at least two tumour types. The following were recorded for each tumour: necrosis in the invasive tumour, invasion of lymphatics or blood vessels, the number of axillary lymph nodes examined and the number of these involved by tumour, tumour size (measured microscopically in tumours up to 20 mm across, and macroscopically in larger tumours). Only ductal carcinomas were graded using the modified Bloom and Richardson method (Elston and Ellis, 1991). The inflammation was assessed in the first surgical specimen in patients undergoing more than one procedure, to avoid inflammation associated with previous surgery. The pattern of inflammatory infiltrate (a) within and (b) at the edge of the invasive tumour was noted as (i) diffuse in the stroma between tumour cells, or focal, (ii) around tumour islands, (iii) around vessels or (iv) around lobules. The intensity of each pattern of inflammatory infiltrate was graded as absent or minimal (0), mild (1), moderate (2) or marked (3). The intensity of inflammation around any carcinoma *in situ*, and the presence of inflammation elsewhere in the specimen was noted.

Comparisons between groups were made with the Mann–Whitney *U*-method, and correlation using Spearman's rank method. The Wilcoxon signed-rank test was used to analyse paired data.

*Immunohistochemistry*

Immunohistochemistry with a panel of antibodies and appropriate pretreatment (Table I) was performed on 21 tumours with a moderate or marked inflammatory infiltrate (12 lobular, eight ductal (one grade I, two grade II and five grade III), one mixed lobular and ductal). A streptavidin–biotin technique was used. Phenol formalin-fixed, paraffin-embedded sections (3 μm) were dried briefly at room temperature then at 56°C for 12–18 h. The sections were dewaxed in xylene, then put into absolute alcohol. Endogenous peroxidase was inhibited with freshly prepared 0.5% hydrogen peroxide in methanol for 10–15 min. Sections were then washed in running tap water for 5 min, and covered with two changes of tris-buffered saline (TBS) pH 7.6 for 5 min each. The sections were then drained and covered with primary antibody (diluted in TBS) for 30 min. After rinsing in TBS, the sections were covered with biotinylated secondary antibody for 30 min (appropriately diluted in TBS, and incorporating 1:25 dilution of normal human serum). After further rinsing with TBS, streptavidin horseradish peroxidase (appropriately diluted in TBS) was applied for 30 min. Then after rinsing with TBS, the sections were covered with freshly prepared diaminobenzidine solution for 10 min. Finally the sections were washed in TBS followed by running tap water for 5 min, counterstained with Mayer's haematoxylin for 2–5 min; rinsed in running tap water, differentiated in 1% acid-alcohol and 'blued' in running tap water. Before application of the primary antibodies to CD3 and von Willebrand factor, swine serum diluted 1:5 was applied for 15 min.

Trypsinisation, if required, was performed before application of the primary antibody using 0.1% trypsin solution (with 0.1% calcium chloride; pH 7.8) at 37°C. Immediately after trypsin treatment the sections were washed in running tap water for 5–10 min. Sections for microwave pretreatment were cut onto Vectabond-coated slides and dried overnight at 56°C. The slides were put in citrate buffer (2.1 g monohydrate citric acid per litre of distilled water, then adjusted to pH 6.0 with 1 N sodium hydroxide) and microwaved for multiples of 7.5 min, topping up the buffer between treatments. After cooling in running tap water for 10 min the sections were put in TBS, then staining started as above.

1B5 gives good staining on frozen sections, but staining on paraffin sections with no pretreatment can be weak. We have obtained good results with 1B5 on paraffin sections with microwave pretreatment. Before this study, to validate staining with microwave pretreatment, we compared it with 1B5 staining on frozen sections, and also with two other antibodies to class II major histocompatibility antigens, WR18 and LN3, on frozen sections. For this purpose frozen and paraffin sections from the following mammary specimens were used: three normal, one lactating, five tumour with adjacent normal breast, one lymphocytic lobulitis. Concordant expression on endothelium, inflammatory cells, lobular and ductal epithelium and tumour was seen. There was stronger staining on frozen sections. There was minimal background staining with WR18, whereas there was a little more with 1B5.

For the tumours in this study the staining with 1B5 (with microwave pretreatment) in the carcinoma and adjacent breast was compared with sections of normal breast [uninvolved quadrants from mastectomies of patients in this series (four), and normal breast included in excision biopsies for benign disease (six)], lactating breast (two) and lymphocytic lobulitis (one).

### Results

The median age of the patients studied was 57 (range 27–87). An axillary clearance was performed in 115 patients with a median of 22 axillary lymph nodes examined (range 8–50). A median of four sections of tumour were examined (range 1–12).

The 123 invasive carcinomas of the breast were typed and graded as follows: Group 1, 55 ductal (15 grade I, 25 grade II, 15 grade III), eight lobular, four tubular or cribriform, five mixed lobular and ductal, one mucoid; and group 2, 38 invasive lobular carcinoma and 12 mixed lobular and ductal carcinomas. In the subsequent analyses invasive tubular and cribriform carcinomas were grouped with invasive ductal grade I.

*Normal breast*

There was minimal inflammation around normal breast ducts and lobules away from the tumour, for example in the quadrant sections in mastectomies.

*Diffuse inflammation associated with tumour*

The predominant pattern of inflammation within and at the edge of tumours was diffuse in the stroma between tumour cells (Table II). In all tumour types the intensity of

**Table II** Intensity of diffuse inflammation within and at the edge of 123 invasive carcinomas

| | *Intensity of inflammation* | | | | | | | |
|---|---|---|---|---|---|---|---|---|
| | *Within tumour* | | | | *Edge of tumour* | | | |
| *Tumour type* | *0* | *1* | *2* | *3* | *0* | *1* | *2* | *3* |
| Ductal grade I | 15 | 4 | | | 13 | 6 | | |
| Ductal grade II | 11 | 8 | 6 | | 9 | 10 | 5 | 1 |
| Ductal grade III | 3 | 5 | 6 | 1 | 1 | 4 | 8 | 2 |
| Lobular | 35 | 10 | 1 | | 30 | 15 | 1 | |
| Mixed lobular and ductal | 9 | 8 | | | 7 | 10 | | |
| Mucinous | 1 | | | | | 1 | | |
| Total | 74 | 35 | 13 | 1 | 60 | 46 | 14 | 3 |

**Table I** Panel of antibodies

| *Antibody* | *Clonality* | *CD* | *Specificity* | *Dilution* | *Pretreatment (min)* | *Source* |
|---|---|---|---|---|---|---|
| CD3 | Poly | CD3 | T cells | 1/100 | Microwave (30) | Dako |
| CD8/144B | Mono | CD8 | T suppressor cells | 1/2 | Microwave (30) | Dr D Mason, Oxford |
| UCHL1 | Mono | CD45RO | Primed T helper cells, macrophages, B cells | Neat | – | Professor PCL Beverley, ICRF London |
| SN130 | Mono | CD45RA | Naive T helper cells, macrophages, B cells | Neat | – | Dr G Janossy, Royal Free Hospital, London |
| L26 | Mono | CD20 | B cells | 1/100 | Microwave (15) | Dako |
| PGM1 | Mono | CD68 | Macrophages | 1/100 | Trypsin (10) | Dako |
| KP1 | Mono | CD68 | Macrophages, granulocytes | 1/100 | Trypsin (10) | Dako |
| TAL-1B5 | Mono | – | α-chain of HLA-DR | Neat | Microwave (15) | ICRF London |
| von Willebrand factor | Poly | – | Endothelium | 1/500 | Trypsin (10) | Dako |
| CAM5.2 | Mono | – | Low molecular weight cytokeratin | 1/10 | Trypsin (10) | ICRF London |

inflammation at the edge was similar to or more than that within the tumour (Wilcoxon statistic 210, $P=0.001$). Diffuse inflammation within the tumour was more marked in the invasive ductal carcinomas than in invasive lobular carcinomas ($P=0.002$, Mann–Whitney $U$). There was increasing intensity of inflammation with increasing grade of ductal carcinoma ($\rho=0.50$, $P=0.00002$). Lobular carcinomas had an intensity of inflammation similar to grade I ductal carcinomas, and significantly less than grade II ($P=0.003$) and grade III ductal carcinomas ($P<0.0001$). Mixed ductal and lobular carcinomas had an intensity of inflammation similar to grade I and II ductal carcinomas, and significantly less than grade III ductal carcinomas ($P=0.005$). Diffuse inflammation at the edge of the tumour and diffuse inflammation within the tumour showed similar relationships with tumour type and grade.

The presence of tumour necrosis was associated with more intense diffuse inflammation within the tumour in ductal carcinoma ($\rho=0.31$, $P=0.009$), but not in mixed carcinoma ($\rho=0.18$) or lobular carcinoma ($\rho=0.18$). The intensity of diffuse inflammation at the tumour edge was associated with the presence of necrosis in lobular carcinoma ($\rho=0.36$, $P=0.007$) as well as in ductal carcinoma ($\rho=0.29$). The proportion of ductal carcinomas with tumour necrosis increased with grade ($\rho=0.51$, $P=0.00002$).

### *Focal inflammation*

Focal inflammation around tumour cells was uncommon. It was seen within only six tumours (two ductal, two lobular and two mixed ductal and lobular), and at the edge of 11 tumours (six ductal, three lobular and two mixed ductal and lobular).

### *Perilobular inflammation*

In all histological tumour types perilobular inflammation was more marked at the edge than within the tumour (Table III; Wilcoxon statistic 570, $P<0.001$). The intensity of perilobular inflammation at the tumour edge was more marked in lobular than ductal carcinomas ($P=0.02$, Mann–Whitney $U$). Perilobular inflammation increased with increasing grade of ductal carcinoma ($\rho=0.28$, $P=0.02$). The intensity of perilobular inflammation at the edge of invasive carcinomas decreased with the age of the patient in ductal carcinomas ($\rho=-0.53$, $P=10^{-5}$), and mixed ductal and lobular carcinomas ($\rho=-0.47$, $P=0.03$), but not in lobular carcinoma ($\rho=-0.08$). When moderate or marked perilobular inflammation was seen in ductal carcinomas there was usually moderate diffuse inflammation within the tumour. By contrast in lobular carcinomas with moderate or marked perilobular inflammation there was usually little or no diffuse inflammation within the tumour (Table IV).

### *Perivascular inflammation*

Perivascular inflammation was more marked at the edge than within tumours (Wilcoxon statistic 1443, $P<0.001$), but was not significantly more common in any histological type of tumour, or in any grade of ductal carcinoma. In ductal carcinomas with moderate or marked perivascular inflammation there was usually moderate diffuse inflammation within the tumour. By contrast in lobular carcinomas with moderate or marked perivascular inflammation there was usually little or no diffuse inflammation within the tumour (see Table V and Figures 1 and 2). The intensity of perilobular inflammation at the tumour edge correlated with the intensity of perivascular inflammation adjacent to the tumour in lobular ($\rho=0.68$, $P<10^{-5}$), but not in ductal ($\rho=-0.07$) or mixed carcinomas ($\rho=0.07$). The combination of moderate or marked perilobular inflammation, with mild, moderate or severe perivascular inflammation was seen in 8 of 46 (17%) lobular carcinomas and 2 of 59 (3%) ductal carcinomas ($P=0.02$, Fisher exact test).

### In situ *carcinoma*

The intensity of inflammation around any *in situ* carcinoma component correlated with the intensity of diffuse inflammation within the invasive component of ductal carcinomas ($\rho=0.50$, $P=0.00005$), and mixed ductal and lobular carcinomas ($\rho=0.54$, $P=0.01$), but not of lobular carcinomas ($\rho=0.08$). In invasive ductal carcinomas the intensity of inflammation around *in situ* carcinoma increased with the grade of the invasive component ($\rho=0.36$, $P=0.003$).

No relationship was found between axillary nodal status and diffuse, perivascular or perilobular inflammation in invasive carcinoma. However, the intensity of inflammation around carcinoma *in situ* correlated with the number of

**Table III** Intensity of perilobular inflammation within and at the edge of 123 invasive carcinomas

| | *Intensity of inflammation* | | | | | | | |
|---|---|---|---|---|---|---|---|---|
| | *Within tumour* | | | | *Edge of tumour* | | | |
| *Tumour type* | *0* | *1* | *2* | *3* | *0* | *1* | *2* | *3* |
| Ductal grade I | 18 | 1 | | | 17 | 2 | | |
| Ductal grade II | 23 | 1 | 1 | | 20 | 3 | 2 | |
| Ductal grade III | 15 | | | | 9 | 3 | 3 | |
| Lobular | 44 | | 2 | | 30 | 7 | 7 | 2 |
| Mixed lobular and ductal | 17 | | | | 14 | 1 | 2 | |
| Mucinous | 1 | | | | | 1 | | |
| Total | 118 | 2 | 3 | | 90 | 17 | 14 | 2 |

**Table IV** Intensity of diffuse inflammation in carcinomas with moderate or marked perilobular inflammation at the edge

| | *Intensity of diffuse inflammation* | | | |
|---|---|---|---|---|
| *Tumour type* | *0* | *1* | *2* | *3* |
| Invasive ductal | | 1 | 4 | |
| Invasive lobular | 5 | 3 | 1 | |

$P=0.01$ (Mann–Whitney)

**Table V** Intensity of diffuse inflammation in carcinomas with moderate or marked perivascular inflammation at the edge

| | *Intensity of diffuse inflammation* | | | |
|---|---|---|---|---|
| *Tumour type* | *0* | *1* | *2* | *3* |
| Invasive ductal | | 2 | 4 | 1 |
| Invasive lobular | 8 | 1 | | |

$P=0.001$ (Mann–Whitney).

**Figure 1** Invasive lobular carcinoma with a circumscribed perivascular cluster of inflammatory cells, but minimal inflammation within the tumour. Haematoxylin and eosin.

involved axillary lymph nodes in ductal carcinoma ($\rho = 0.42$, $P = 0.0008$) especially grade III ($\rho = 0.81$, $P = 0.0003$), but not in lobular carcinoma ($\rho = 0.004$).

*Immunohistochemistry*

In the normal breast there was minimal inflammation, with small numbers of T cells (including $CD8^+$) and macrophages around and within the epithelium of lobules and ducts. Only a very occasional B cell was seen within the lobular epithelium.

The diffuse inflammation within tumours was predominantly composed of T cells and macrophages. There tended to be more macrophages than T cells, particularly in lobular carcinoma. Only small numbers of B cells were present. $CD45RO^+$ cells usually outnumbered $CD45RA^+$ cells. The perilobular and perivascular inflammation by contrast was composed of similar numbers of B and T cells, with few macrophages (see Figure 2). There were similar numbers of $CD45RO^+$ and $CD45RA^+$ cells.

**Figure 2** Invasive ductal carcinoma showing inflammation both within the tumour and around an adjacent lobule. (**a**) There are T cells (CD3) both within the tumour and around the adjacent lobule. (**b**) B cells (CD20) are largely confined to around the lobule. (**c**) Macrophages (CD68) are seen within the tumour and to a lesser extent around the adjacent lobule. All the same magnification.

Expression of HLA-DR was assessed in ten sections of normal non-lactating breast: 1B5 stained some lymphocytes in ductal and lobular epithelium, and vessels adjacent to lobules and ducts (confirmed with staining for leucocyte common antigen and von Willebrand factor). In three biopsies there was weak epithelial staining (patchy staining of inner epithelial cells), and in one further biopsy there was stronger epithelial staining (all epithelial cells in lobule or duct positive). In addition, both biopsies of lactating breast showed uniform strong epithelial staining. In the one biopsy of lymphocytic lobulitis there was patchy epithelial staining, weak in some areas and strong in others. There was staining of normal lobules and ducts adjacent to all 19 tumours where normal breast was present (8 weak, 11 strong) see Figure 3. Epithelial expression of HLA-DR was sometimes, but not always, associated with perilobular inflammation.

### Discussion

The role of inflammation in tumours is controversial. Studies of the prognostic significance of inflammation in carcinoma of the breast, even with multivariate analysis, have produced conflicting results. A potential explanation of this is that few

**Figure 3** HLA-DR expression shown with 1B5 is seen (**a**) in vessels and around some acini of the normal lobule. (**b**) It is present weakly on the ductal carcinoma cells, and more strongly on inflammatory cells within the tumour, and on the adjacent lobules. Both the same magnification.

previous studies have looked at the pattern of inflammation in tumours.

In invasive carcinomas we found that diffuse inflammation in the stroma between tumour cells was the predominant pattern of inflammation. Most studies do not distinguish between different patterns of inflammation and use a general score, which probably in most tumours is similar to what we describe as diffuse inflammation. We found more intense inflammation in ductal than lobular carcinoma in agreement with the only large series that looked at inflammation in different histological types (Aaltoma *et al.*, 1992). Increasing inflammation with increasing histological grade has also been found by others (Aaltoma *et al.*, 1992; Black *et al.*, 1975; Elston *et al.*, 1982; Fisher *et al.*, 1983; Kurtz *et al.*, 1990). Most, but not all, studies have noted an association between inflammation and necrosis (Fisher *et al.*, 1975; Black *et al.*, 1975; Fisher *et al.*, 1983; Aaltoma *et al.*, 1992; An *et al.*, 1987). We found diffuse inflammation, histological grade and necrosis were interrelated in ductal carcinomas, but the correlation between the diffuse inflammation and necrosis was weak ($\rho = 0.31$) so there must be factors in addition to necrosis causing the inflammation. The reported associations of inflammation with tumour expression of c-erbB-2 (Rilke *et al.*, 1991) and colony-stimulating factor 1 (Scholl *et al.*, 1994) are of potential interest. Immunohistochemistry showed that the diffuse inflammation in tumours was predominantly composed of T cells and macrophages. The majority of other studies have found that T cells outnumbered macrophages (An *et al.*, 1987; Bhan and DesMarais, 1983; Giorno, 1983; Hurliman *et al.*, 1985; von Kleist, 1987; Zuk and Walker, 1987), but a significant minority, like us, found more macrophages than T cells (Gottlinger *et al.*, 1985; Horny *et al.*, 1986; van Ravenswaay *et al.*, 1992). Few B cells and few or no natural killer cells are consistent findings in the literature.

Focal inflammation close to and within tumour nests was uncommon in all tumour types in agreement with other series (Bhan *et al.*, 1983; Gottlinger *et al.*, 1985; Lwin *et al.*, 1985). It has been suggested that this low frequency of inflammatory cells close to the tumour cells indicates that the lymphocytes and macrophages do not represent an immune response to the tumour cells.

Perilobular inflammation was rarely seen within the tumour, probably owing to destruction of normal structures by the tumour. Black and Speer (1955) found marked inflammation in the tumour was associated with perilobular inflammation. They made no comment on histological tumour types, but the pattern described is similar to our findings in ductal carcinomas. By contrast in lobular carcinomas with perilobular inflammation there was usually little or no diffuse inflammation within the tumour. Immunohistochemistry showed that, in contrast to the diffuse inflammation, the perilobular inflammation was predominantly composed of B and T cells with few macrophages.

The relative absence of perivascular inflammation within the tumour may be because of differences between vessels within the tumour and normal vessels at the edge. There is evidence of differences between the endothelium in mammary carcinomas and in normal breast (Hagemeier *et al.*, 1986; Wang *et al.*, 1993). Perivascular inflammation in breast cancer has been little studied. Black *et al.* (1975) found perivenous inflammation was associated with poorly differentiated tumours and longer survival in univariate analysis. It has been suggested that perivascular inflammation is a reflection of cell-mediated immunity by analogy with the changes in delayed type hypersensitivity reactions in the skin (Dvorak *et al.*, 1981). The perivascular inflammation, like the perilobular inflammation, was predominantly composed of B and T cells with few macrophages.

Normal breast epithelium shows no expression of HLA-DR (Bartek *et al.*, 1987; Lucin *et al.*, 1994), except during late pregnancy and lactation (Bartek *et al.*, 1987). We found consistent staining for HLA-DR in lobules adjacent to carcinomas, in agreement with Bartek *et al.* (1987), who found focal staining for HLA-DR in normal epithelium adjacent to 70% of carcinomas. Lucin *et al.* (1994), however, found no such staining of the normal breast 'in the vicinity' of carcinomas. Epithelial expression of HLA-DR was in some tumours associated with perilobular inflammation.

A combination of perivascular and perilobular inflammation mimicking lymphocytic lobulitis was much more common in lobular (17%) than ductal carcinomas (3%). As well as the distribution of the inflammation, the immunophenotype of the inflammatory cells (B and T cells) and epithelium (HLA-DR expression) are similar to lymphocytic lobulitis. Lymphocytic lobulitis is thought to be an autoimmune disorder, but the mechanism of the inflammatory changes is not understood. The fact that in mammary carcinoma perilobular and perivascular inflammation were almost always only seen within or adjacent to the carcinoma, and that similar patterns may be seen associated with metastases (Lee *et al.*, 1995) suggest that the inflammation is related to the tumour. In ductal carcinomas with perivascular or perilobular inflammation there was usually moderate diffuse inflammation within the tumour. By contrast, in lobular carcinomas with perivascular or perilobular inflammation there was usually little or no diffuse inflammation within the tumour.

The intensity of inflammation around the carcinoma *in situ* correlated with the diffuse inflammation within invasive ductal carcinomas and with the grade of the invasive ductal carcinoma. Black *et al.* (1975) also found a correlation between the inflammation around the *in situ* and invasive carcinoma. This is consistent with more intense inflammation in high-grade ductal carcinoma *in situ* (Ramachandra *et al.*, 1990) as in invasive ductal carcinoma, and that the grade of *in situ* and infiltrating components correlate with each other (Lampejo *et al.*, 1994).

There are conflicting results from studies of inflammation and tumour stage (Aaltoma *et al.*, 1992; Lucin *et al.*, 1994; Lauder *et al.*, 1977; Syrjanen *et al.*, 1978; Bhan and DesMarais, 1983). We did not find any relationship between inflammation within or at the edge of the invasive tumour and stage, but the intensity of inflammation around carcinoma *in situ* correlated with the number of involved axillary lymph nodes in ductal carcinomas, particularly grade III. This is difficult to explain and may be a chance result.

Some studies have found more inflammation in the tumours of younger women (Fisher *et al.*, 1975; Kurtz *et al.*, 1990). We found less perilobular inflammation in older women with ductal and mixed ductal and lobular carcinomas, consistent with lobular atrophy, but interestingly this pattern was not seen in lobular carcinoma. Other patterns of inflammation were not related to age.

In conclusion, we have found different patterns of inflammation composed of different cell types with different frequencies in ductal and lobular carcinomas. They may have different functional significance. The conflicting findings in prognostic studies of inflammation in carcinoma of the breast may in part be due to failure to recognise these different patterns of inflammation. There is increasing interest in immunotherapy as a treatment for carcinoma of the breast, and an understanding of inflammation, including the significance of the different patterns, in mammary carcinoma is potentially important.

### References

AALTOMA S, LIPPONEN P, ESKELINEN M, KOSMA V-M, MARIN S, ALHAVA E AND SYRJANEN K. (1992). Lymphocyte infiltrates as a prognostic variable in female breast cancer. *Eur. J. Cancer*, **28A**, 859–864.

ALDERSON MR, HAMLIN I AND STAUNTON MD. (1971). The relative significance of prognostic factors in breast carcinoma. *Br. J. Cancer*, **25**, 646–656.

AN T, SOOD U, PIETRUK T, CUMMINGS G, HASHIMOTO K AND CRISSMAN JD. (1987). In situ quantitation of inflammatory mononuclear cells in ductal infiltrating breast carcinoma. Relation to prognostic factors. *Am. J. Pathol.*, **128**, 52–60.

AZZOPARDI JG. (1979). *Problems in Breast Pathology*. London: WB Saunders.

BALCH CM, RILEY LB, BAE YJ, SALMERON MA, PLATSOUCAS CD, VON ESCHENBACH A AND ITOH K. (1990). Patterns of human tumor-infiltrating lymphocytes in 120 human cancers. *Arch. Surg.*, **125**, 200–205.

BARTEK J, PETREK M, VOJTESEK B, BARTKOVA J, KOVARIK J AND REJTHAR A. (1987). HLA-DR antigens on differentiating human mammary gland epithelium and breast tumours. *Br. J. Cancer*, **56**, 727–733.

BHAN AK AND DESMARAIS CL. (1983). Immunohistologic characterisation of major histocompatibility antigens and inflammatory cellular infiltrate in human breast cancer. *J Natl Cancer Inst.*, **71**, 507–516.

BLACK MM AND SPEER FD. (1955). Periductal lymphoid infiltrations in mammary tissue. *Arch. Pathol.*, **60**, 457–461.

BLACK MM, BARCLAY THC AND HANKEY BF. (1975). Prognosis in breast cancer utilizing histologic characteristics of the primary tumor. *Cancer*, **36**, 2048–2055.

CHETTY R AND BUTLER AE. (1993). Lymphocytic mastopathy associated with infiltrating lobular breast carcinoma. *J. Clin. Pathol.*, **46**, 376–377.

DVORAK HF, DICKERSIN GR, DVORAK AM, MANSEAU EJ AND PYNE K. (1981). Human breast carcinoma: fibrin deposits and desmoplasia. Inflammatory cell type and distribution. Microvasculature and infarction. *J. Natl Cancer Inst.*, **67**, 335–345.

ELSTOW CW AND ELLIS IO. (1991). Pathological prognostic factors in breast cancer. I. The value of histological grade in breast cancer: experience from a large study with long-term follow-up. *Histopathology*, **19**, 403–410.

ELSTON CW, GRESHAM GA, RAO GS, ZEBRO T, HAYBITTLE JL, HOUGHTON J AND KEARNEY G. (1982). The Cancer Research Campaign (King's/Cambridge) trial for early breast cancer: clinico-pathological aspects. *Br. J. Cancer*, **45**, 655–669.

FISHER ER, GREGORIO RM, FISHER B, REDMOND C, VELLIOS F, SOMMERS SC AND COOPERATING INVESTIGATORS. (1975). The pathology of invasive breast cancer. A syllabus derived from findings of the National Surgical Adjuvant Breast Project (Protocol No. 4). *Cancer*, **36**, 1–85.

FISHER ER, KOTWAL N, HERMANN C, FISHER B AND CONTRIBUTING INVESTIGATORS OF THE NATIONAL SURGICAL ADJUVANT BREAST PROJECT. (1983). Types of tumor lymphoid response and sinus histiocytosis. Relationship to five-year, disease-free survival in patients with breast cancer. *Arch. Pathol. Lab. Med.*, **107**, 222–227.

GIORNO R. (1983). Mononuclear cells in malignant and benign human breast tissue. *Arch. Pathol. Lab. Med.*, **107**, 415–417.

GOTTLINGER HG, RIEBER P, GOKEL JM, LOHE KJ AND RIETHMULLER G. (1985). Infiltrating mononuclear cells in human breast carcinoma: predominance of T4+ monocytic cells in the tumor stroma. *Int. J. Cancer*, **35**, 199–205.

HAGEMEIER H-H, VOLLMER E, GOERDT S, SCHULZE-OSTHOFF K AND SORG C. (1986). A monoclonal antibody reacting with endothelial cells of budding vessels in tumors and inflammatory tissues, and non-reactive with normal adult tissues. *Int. J. Cancer*, **38**, 481–488.

HORNY H-P AND HORST H-A. (1986). Lymphoreticular infiltrates in invasive ductal breast cancer. *Virchows. Arch. A Pathol. Anat.*, **409**, 275–286.

HURLIMANN J AND SARAGA P. (1985). Mononuclear cells infiltrating human mammary carcinomas: immunohistochemical analysis with monoclonal antibodies. *Int. J. Cancer*, **35**, 753–762.

KURTZ JM, JACQUEMIER J, AMALRIC R, BRANDONE H, AYME Y, HANS D, BRESSAC C AND SPITALIER J-M. (1990). Why are local recurrences after breast-conserving therapy more frequent in younger patients? *J. Clin. Oncol.*, **8**, 591–598.

LAMMIE GA, BOBROW LG, STAUNTON MDM, LEVISON DA, PAGE G AND MILLIS RR. (1991). Sclerosing lymphocytic lobulitis of the breast – evidence for an autoimmune pathogenesis. *Histopathology*, **19**, 13–20.

LAMPEJO OT, BARNES DM, SMITH P AND MILLIS RR. (1994). Evaluation of infiltrating ductal carcinomas with a DCIS component: correlation of the histologic type of the in situ component with grade of the infiltrating component. *Semin. Diagn. Pathol.*, **11**, 215–222.

LAUDER I, AHERNE W, STEWART J AND SAINSBURY R. (1977). Macrophage infiltration of breast tumours: a prospective study. *J. Clin. Pathol.*, **30**, 563–568.

LEE AHS, MILLIS RR AND BOBROW LG. (1996). Lymphocytic lobulitis and carcinoma of the breast. (letter). *Histopathology*, **28**, 94–95.

LUCIN K, ITERNICKE Z AND JONJIC N. (1994). Prognostic significance of T-cell infiltrates, expression of $\beta$2-microglobulin and HLA-DR antigens in breast carcinoma. *Pathol. Res. Pract.*, **190**, 1134–1140.

LWIN KY, ZUCCARINI O, SLOANE JP AND BEVERLEY PCL. (1985). An immunohistochemical study of leukocyte localisation in benign and malignant breast tissue. *Int. J. Cancer*, **36**, 433–438.

MIESCHER S, WHITESIDE TL, CARREL S AND VON FLIEDNER V. (1986). Functional properties of tumor-infiltrating and blood lymphocytes in patients with solid tumors: effects of tumor cells and their supernatants on proliferative responses of lymphocytes. *J. Immunol.*, **136**, 1899–1907.

PARL FF AND DUPONT WD. (1982). A retrospective cohort study of histologic risk factors in breast cancer patients. *Cancer*, **50**, 2410–2416.

RAMACHANDRA S, MACHIN L, ASHLEY S, MONAGHAN P AND GUSTERSON BA. (1990). Immunohistochemical distribution of c-erbB-2 in in situ breast carcinoma – a detailed morphological analysis. *J. Pathol.*, **161**, 7–14.

RILKE F, COLNAGHI MI, CASCINELLI N, ANDREOLA S, BALDINI MT, BUFALINO R, DELLA PORTA G, MENARD S, PIEROTTI MA AND TESTORI A. (1991). Prognostic significance of HER-2/neu expression in breast cancer and its relationship to other prognostic factors. *Int. J. Caner*, **49**, 44–49.

ROSES DF, BELL DA, FLOTTE TJ, TAYLOR R, RATECH H AND DUBIN N. (1982). Pathologic predictors of recurrence in stage 1 (T1NOMO) breast cancer. *Am. J. Clin. Pathol.*, **78**, 817–820.

SCHOLL SM, PALLUD C, BEUVON F, HACENE K, STANLEY ER, ROHRSCHNEIDER L, TANG R, POUILLART P AND LIDEREAU R. (1994). Anti-colony-stimulating factor-1 antibody staining in primary breast adenocarcinomas correlates with marked inflammatory cell infiltrates and prognosis. *J. Natl Cancer Inst.*, **86**, 120–126.

SCHWARTZ IS AND STRAUCHEN JA. (1990). Lymphocytic mastopathy. An autoimmune disease of the breast? *Am. J. Clin. Pathol.*, **93**, 725–730.

SYRJANEN KJ AND HJELT LH. (1978). Tumor–host interrelationships in carcinoma of the female breast. *Surg. Gynecol. Obstet.*, **147**, 43–48.

UNDERWOOD JCE. (1974). Lymphoreticular infiltration in human tumours: prognostic and biological implications: a review. *Br. J. Cancer*, **30**, 538–547.

VAN RAVENSWAAY CLAASEN HH, KLUIN PM AND FLEUREN GJ. (1992). Tumor infiltrating cells in human cancer. On the possible role of $CD16^+$ macrophages in antitumor cytotoxicity. *Lab. Invest.*, **67**, 166–174.

VON KLEIST S, BERLING J, BOHLE W AND WITTEKIND C. (1987). Immunohistochemical analysis of lymphocyte subpopulations infiltrating breast carcinomas and benign lesions. *Int. J. Cancer*, **40**, 18–23.

WANG JM, KUMAR S, PYE D, VAN AGTHOVEN AJ, KRUPINSKI J AND HUNTER RD. (1993). A monoclonal antibody detects heterogeneity in vascular endothelium of tumours and normal tissues. *Int. J. Cancer*, **54**, 363–370.

ZUK JA AND WALKER RA. (1987). Immunohistochemical analysis of HLA antigens and mononuclear infiltrates of benign and malignant breast. *J. Pathol.*, **152**, 275–285.